# Role of Th17 Cytokines in the Liver’s Immune Response during Fatal Yellow Fever: Triggering Cell Damage Mechanisms

**DOI:** 10.3390/cells11132053

**Published:** 2022-06-28

**Authors:** Marcos Luiz Gaia Carvalho, Luiz Fábio Magno Falcão, Jeferson da Costa Lopes, Caio Cesar Henriques Mendes, Fábio Alves Olímpio, Vanessa do Socorro Cabral Miranda, Lais Carneiro dos Santos, Daniel Dias Pinheiro de Moraes, Marcos Virgilio Bertonsin Filho, Luccas Delgado da Costa, Raimunda do Socorro da Silva Azevedo, Ana Cecília Ribeiro Cruz, Vanessa Costa Alves Galúcio, Lívia Caricio Martins, Maria Irma Seixas Duarte, Arnaldo Jorge Martins Filho, Jorge Rodrigues de Sousa, Pedro Fernando da Costa Vasconcelos, Juarez Antônio Simões Quaresma

**Affiliations:** 1 Instituto Evandro Chagas, Secretaria de Vigilância em Saúde, Ministério da Saúde, Ananindeua 67030-000, PA, Brazil; marcosgaia@outlook.com (M.L.G.C.); jefersonchaz@hotmail.com (J.d.C.L.); vanessacabralmiranda@gmail.com (V.d.S.C.M.); laiscarneiros@gmail.com (L.C.d.S.); raimundaazevedo@iec.gov.br (R.d.S.d.S.A.); anacecilia@iec.gov.br (A.C.R.C.); liviamartins@iec.gov.br (L.C.M.); arnaldofilho@iec.gov.br (A.J.M.F.); krekrodrigues@gmail.com (J.R.d.S.); 2 Centro de Ciências Biológicas e da Saúde, Universidade do Estado do Pará, Belém 66087-662, PA, Brazil; fabiofalcao@uepa.br (L.F.M.F.); caio_henriques12@hotmail.com (C.C.H.M.); 3 Núcleo de Medicina Tropical, Universidade Federal do Pará, Belém 66055-240, PA, Brazil; f.olimpiomilitar@gmail.com (F.A.O.); pinheiro.daniel95@gmail.com (D.D.P.d.M.); marcosvirgilo@hotmail.com (M.V.B.F.); luccasdelgado10@gmail.com (L.D.d.C.); 4 Curso de Biomedicina, Faculdade Cosmopolita, Belém 66615-005, PA, Brazil; vanessagalucio@gmail.com; 5 Faculdade de Medicina, Universidade de São Paulo, São Paulo 01246-903, SP, Brazil; miduarte@usp.br

**Keywords:** yellow fever, Th17 profile, cell damage, liver immune response

## Abstract

Yellow fever (YF) is an infectious and acute viral haemorrhagic disease that triggers a cascade of host immune responses. We investigated the Th17 cytokine profile in the liver tissue of patients with fatal YF. Liver tissue samples were collected from 26 deceased patients, including 21 YF-positive and 5 flavivirus-negative patients, with preserved hepatic parenchyma architecture, who died of other causes. Histopathological and immunohistochemical analysis were performed on the liver samples to evaluate the Th17 profiles (ROR-γ, STAT3, IL-6, TGF-β, IL-17A, and IL-23). Substantial differences were found in the expression levels of these markers between the patients with fatal YF and controls. A predominant expression of Th17 cytokine markers was observed in the midzonal region of the YF cases, the most affected area in the liver acinus, compared with the controls. Histopathological changes in the hepatic parenchyma revealed cellular damage characterised mainly by the presence of inflammatory cell infiltrates, Councilman bodies (apoptotic cells), micro/macrovesicular steatosis, and lytic and coagulative necrosis. Hence, Th17 cytokines play a pivotal role in the immunopathogenesis of YF and contribute markedly to triggering cell damage in patients with fatal disease outcomes.

## 1. Introduction

Yellow fever (YF) is an arthropod-borne viral disease with a high fatality rate in tropical endemic areas of Africa and South America [1,2]. The disease is caused by the YF virus (YFV), a single-stranded positive-sense RNA virus belonging to the *Flaviviridae* family (genus, *Flavivirus*), whose genome encodes three structural proteins (C, M, and E) and seven non-structural proteins (NS1, NS2A, NS2B, NS3, NS4A, NS4B, and NS5) [3,4]. YF is a viral haemorrhagic fever that causes viral sepsis. In severe cases, YF can lead to dysfunction and/or failure of several organs, including the liver, kidney, lung, heart, spleen, and intestines. Other manifestations of the severe form of this disease include vascular impairment, severe liver damage, and haemorrhage [5,6,7].

In recent years, there has been an increase in the incidence of YFV infection leading to the occurrence of outbreaks and epidemics. This is attributed to the ease of transmission of the virus. In Brazil, from 2016 to 2019, the disease re-emerged in cities with a high population density, leading to an increase in epizootics and confirmed human YF cases, placing a high burden on healthcare systems [8,9,10].

The immunopathogenesis of YFV infection-induced liver disease is speculated to promote cell injury in the host cells. The immune response is characterised by severe tissue damage in the midzonal area of the liver, leading to hepatocyte steatosis, followed by apoptosis and necrosis of the infected cells. This is thought to be due to the action of Kupffer cells, dendritic cells, T lymphocytes (Th1 (Th-T helper lymphocytes) and Th2), and NK (natural killer) cells, which trigger the production of cytokines and enzymes that exacerbate oxidative stress and worsen cell damage [11,12,13].

YF is a systemic and a fulminant disease and triggers the immune system, leading to an enhanced proinflammatory response mediated by a variety of T helper lymphocyte subsets (Th1 and Th2 lymphocytes) [14,15]. Quaresma et al. described an important participation of TCD4+ Th1 lymphocytes, accompanied by the activity of TCD8+ cells, NK cells, macrophages, and B lymphocytes [11,12,14]. TCD4+ Th17 lymphocytes are involved in the recruitment of leukocytes, mainly neutrophils, and the induction of inflammation. Cytokines such as IL-6 and IL-1 stimulate the differentiation of TCD4+ cells to the Th17 subgroup, with IL-23 being important in the proliferation and maintenance of these cells. Furthermore, cytokines such as IL-6 and TGF-β and transcription factors such as STAT3 (STAT—signal transducers and activators of transcription) and ROR-γ (retinoic acid related orphan receptor γ) are involved in the processes of Th17 cell formation and differentiation. IL-17 is one of the main cytokines produced by this subgroup of TCD4+ lymphocytes and has the ability to induce acute inflammation and stimulate the production of antimicrobial substances [16,17,18].

In general, the Th17 profile is an important factor in the pathogenesis of several infections and is involved in the immune response to viral, bacterial, and fungal agents [19,20,21]. In dengue virus infection, the Th17 profile was reported to be involved in the pathophysiology of haemorrhagic forms of dengue disease [22]. However, studies on the role of Th17 cytokines, particularly in severe forms of YF disease, are still scarce. One of the characteristics of severe forms of YF is the intense inflammation that can lead to vascular and organ involvement, such as the liver. Thus, as in severe dengue, would the Th17 profile play an important role in the pathogenesis of the disease, contributing to the vascular alteration and liver damage observed in severe forms of YF?

Thus, the identification of immune factors involved in the immunopathogenesis of YF is important for understanding the evolution of the infection, the identification of possible therapeutic targets, and the development of more effective vaccines without severe adverse effects. In this study, we therefore aimed to evaluate the in situ Th17 cytokine profiles of patients with fatal YF.

## 2. Methods

### 2.1. Study Design

An analytical cross-sectional study was carried out using liver samples from the biobank of the Section of Pathology at the Evandro Chagas Institute of the Ministry of Health of Brazil, from 2000 to 2016. Liver samples obtained by viscerotomy from patients who died from YF were included in the sample of this work. The diagnosis of YFV infection was confirmed by histopathology, immunohistochemistry against the viral antigen, and Real-time quantitative RT-PCR (RT-qPCR) analysis [6].

### 2.2. Patients, Samples, and Diagnosis of YFV Infection

Samples were obtained from the archives of the Department of Pathology, Evandro Chagas Institute (Belem, Brazil). No comorbidities were recorded on the case notification forms from all 21 patients included in this study. In addition, the demographic data showed that the age of the investigated samples ranged between 3 and 63 years, and the majority were male (66.6%). More detailed information about the subjects included in this study can be found in Table 1. The confirmation of the diagnosis for the positive cases of YF was based on the study by Olimpio et al. [6], including histopathological, immunohistochemical, and Real-time quantitative RT-PCR (RT-qPCR) analysis.

Additionally, we obtained liver tissue samples from five deceased patients (between 20 and 50 years old, four male and one female) who had a non-infectious or inflammatory liver disease and showed no histopathological changes or signs of infectivity with other hepatotropic viruses, based on the records from the death verification service in the city of Belém in the state of Pará, Brazil.

Liver samples were obtained by viscerotomy. The samples were then fixed in 10% buffered formalin, embedded in paraffin, and sectioned into 5 μm thick sections using a microtome. The sections were stained using haematoxylin–eosin (H&E), reticulin, Masson trichrome, and Perls staining to assess morphological changes. Additional 5 μm thick sections were obtained and mounted on salinized slides for subsequent immunohistochemical staining for specific immune markers.

### 2.3. Histology and Semi-Quantitative Analysis

Histological analysis was performed using an Axio Imager Z1 microscope (Zeiss, Oberkochen, Germany) model 456,006 (400× magnification).

Each zone of Rappaport’s hepatic acinus (Z1: Periportal zone, Z2: Midzonal zone, and Z3: Pericentral zone) and portal tract (PT) was evaluated using a semi-quantitative scale from 0 to 4 (0: absent, 1: mild, 2: moderate ++, 3: intense +++, 4: very intense ++++) to classify the extent of tissue damage [12]. Histopathologic aspects were evaluated semi-quantitatively by randomly selecting ten fields in the hepatic parenchyma of the patients with fatal YF or controls for analyses at a high magnification. Each field was subdivided into 10 × 10 areas delimited by a 0.0625 mm^2^ grid [6].

### 2.4. Immunohistochemistry

Immunostaining of the hepatic tissues with antibodies specific for ROR-γ (Abcam, Cambridge, UK, ab 219,496, dilution 1:100), STAT3 (Abcam, Cambridge, UK, ab 76,315, dilution 1:100), IL-6 (Abcam, Cambridge, UK, ab 6672, dilution 1:100), TGF-β (Abcam, Cambridge, UK, ab 190,503, dilution 1:100), IL-17 (Abcam, Cambridge, UK, ab 79,056, dilution 1:100), and IL-23 (Abcam, Cambridge, UK, ab 45,420, dilution 1:100) was performed using the biotin–streptavidin–peroxidase method [6]. Briefly, the tissue samples were dewaxed in xylol and hydrated in ethyl alcohol at concentrations of 90%, 80%, and 70%. Endogenous peroxidase was blocked with 3% hydrogen peroxide (H_2_O_2_) for 45 min. Antigens were retrieved by incubation in citrate buffer (pH 6.0) for 20 min at 90 °C. Non-specific proteins were blocked by incubating the sections in 10% skim milk for 30 min.

The primary antibodies were diluted in 1% bovine serum albumin solution (BSA) (Sigma Aldrich, Saint Louis, MA, USA) for 14 h, and the secondary biotinylated antibody, LSAB, (DakoCytomation, Glostrup, Denmark) was then added and incubated for 30 min at 37 °C. For visualisation, the specimens were treated with a chromogenic solution composed of 0.03% diaminobenzidine and 3% hydrogen peroxide. Histological sections were washed in distilled water, counterstained with Harris haematoxylin for 1 min, dehydrated in ethanol (70%, 80%, and 90%), and deparaffinised in xylene.

### 2.5. Quantitative Analysis and Photo-Documentation

The markers used to characterise the in situ Th17 profile were visualised using an Axio Imager Z1 microscope (Zeiss). Cellular immunostaining results were evaluated quantitatively by randomly selecting ten fields under microscope in the hepatic parenchyma of the patients with fatal YF or controls for analyses at a high magnification (400×). Each field was subdivided into 10 × 10 areas delimited by a 0.0625 mm^2^ grid [6].

### 2.6. Statistical Analysis

The data were stored in a Microsoft Excel 2007 spreadsheet and analysed using GraphPad Prism 5.0 (San Diego, CA, USA). The numerical variables were expressed as the mean, median, standard deviation, and variance. One-way ANOVA and Tukey’s test were performed; results were considered statistically significant at *p* < 0.05 [6,11,12,13].

### 2.7. Ethical Aspects

This study was conducted following relevant guidelines and regulations of the Ministry of Health Ethics Committee (CONEP, Ottawa, ON, Canada). In addition, all experimental protocols carried out in this study were approved by the Research Ethics Committee of the Evandro Chagas Institute (IEC) (number 2.462.701), Rodovia BR-316, km-07, Ananindeua, Pará, Brazil. The study followed the recommendations in the resolution of the National Health Council No. 466/2012, and the principles of the Declaration of Helsinki. Written informed consent was waived by the IEC Ethics Committee (decision number 2.462.701) since all the liver samples were obtained from deceased individuals.

### 2.8. Availability of Data and Materials

The database used and/or analysed during the current study is not publicly accessible but can be available, upon reasonable request, from the corresponding authors.

## 3. Results

### 3.1. Histopathological Analysis

**Figure 1:**
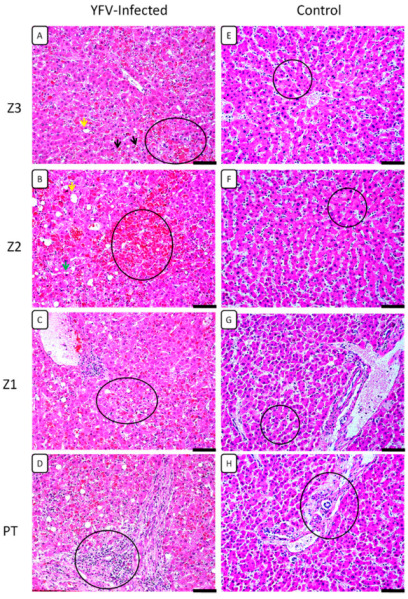
Histopathological analysis in the zones Z3, Z2, and Z1 and PT in the hepatic parenchyma of patients with fatal YF and normal controls. (**A**) Macrovesicular steatosis (yellow arrow), Councilman bodies (black arrow), coagulative necrosis (circle). (**B**) Macrovesicular steatosis (yellow arrow), microvesicular steatosis (green arrow), intense haemorrhagic necrosis (circle). (**C**) Mild coagulative necrosis and steatosis (circle). (**D**) Presence of inflammatory infiltrates in the PT (circle). (**E**–**H**) Preservation of the hepatic parenchyma (Z3, Z2, Z1, and PT) in the control cases (circles). Magnification: 400× (scale bar, 20 µm). Z3: Pericentral zone; Z2: Midzonal zone; Z1: Periportal zone; PT: Portal tract. (Patient 03).

Histopathological changes in the liver were characterised by the presence of evident midzonal (Z2) lesions, consisting of hepatocytes with changes ranging from swelling, macro- and microvesicular steatosis, production of Councilman bodies (apoptotic cells), and to a lesser or greater extent, lytic and coagulative necrosis (Table 2, Figure 1). Other changes observed in both the acinus zones (Z1, Z2, and Z3) and the portal tract (PT) are presented in Table 2, highlighting the presence of hyperplasia and hypertrophy of Kupffer cells, sinusoidal congestion, acinar haemorrhage, and changes in vascular structures in the central lobular vein zone (Z3) and in the PT. These changes were accompanied by the infiltration of lymphocytes, plasma cells, and neutrophils surrounding the acinar region. The infiltrates were found especially around the foci of moderate-intensity lytic necrosis, congestion, haemorrhage, and tissue damage (Table 2).

### 3.2. Analysis of the Response to the Th17 Profile in the Hepatic Parenchyma in Cases with Fatal YF

**Figure 2:**
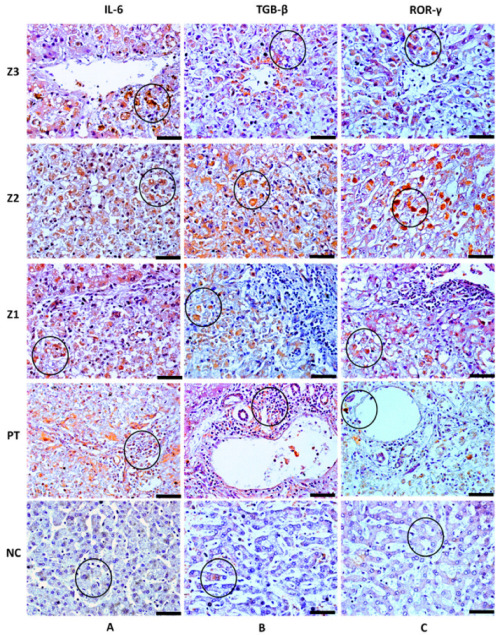
Positive immunohistochemistry staining for IL-6, TGF-β, and ROR-γ in the zones Z3, Z2, and Z1 and PT in the liver parenchyma of patients with fatal YF and normal controls. (**A**) Immunolabeling for IL-6 (circle) in Kupffer cells (**A-Z3**), hepatocytes (**A-Z2**,**A-Z1**), and the inflammatory infiltrate (**A-PT**). (**B**) Immunolabeling for TGF-β (circle) in hepatocytes (**B-Z3**,**B-Z2**,**B-Z1**) and in the inflammatory infiltrate (**B-PT**). (**C**) Immunolabeling for ROR-γ (circle) in hepatocytes (**C-Z3**,**C-Z2**,**C-Z1**) and T cells (**C-PT**). Absence of labelling for IL-6 and preservation of the liver parenchyma (**A-NC**), slight labelling for TGF-β and ROR-γ in Z2, and preservation of the liver parenchyma (**B-NC**,**C-NC**). Magnification: 400× (scale bar, 20 µm). Z3: Pericentral zone; Z2: Midzonal zone; Z1: Periportal zone; PT: Portal tract. (Patient 03).

**Figure 3:**
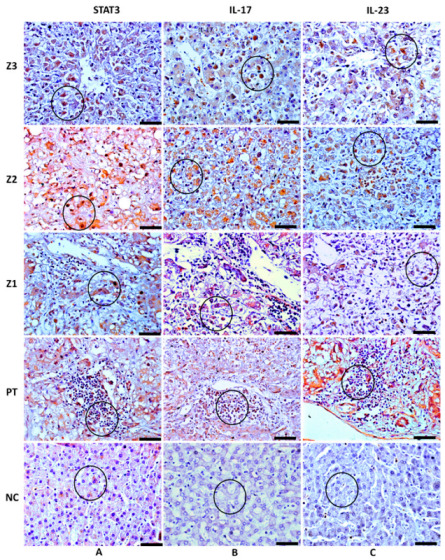
STAT3, IL-17A, and IL-23 positive immunohistochemistry staining in zones Z3, Z2, and Z1 and PT in the liver parenchyma of patients with fatal YF and normal controls. (**A**) Immunolabeling for STAT3 in hepatocytes (circle) (**A-Z3**,**A-Z2**,**A-Z1**) and in the inflammatory infiltrate (**A-PT**). (**B**) Immunolabeling for IL-17A in hepatocytes (circle) (**B-Z3**,**B-Z2**,**B-Z1**), and in the inflammatory infiltrate (**B-PT**). (**C**) Immunolabeling for IL-23 in hepatocytes (circle) (**C-Z3**,**C-Z2**,**C-Z1**) and inflammatory infiltrate in (**C-PT**). Light marking for STAT3 in Z2 and preservation of the hepatic parenchyma (Circle) (**A-NC**), absence of marking for IL-17A and IL-23, and preservation of the hepatic parenchyma (circle) (**B-NC**,**C-NC**). Magnification: 400× (scale bar, 20 µm)**.** Z3: Pericentral zone; Z2: Midzonal zone; Z1: Periportal zone; PT: Portal tract. (Patient 03).

**Figure 4:**
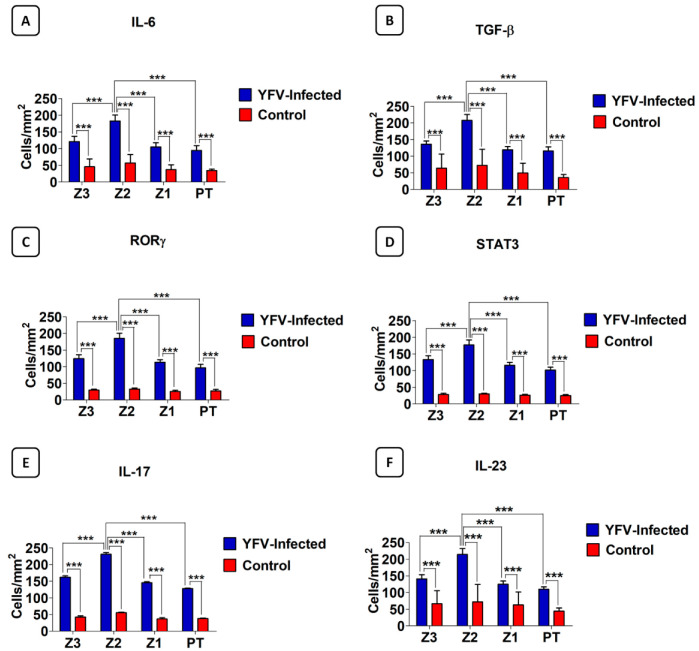
Quantitative analysis for IL-6 (**A**), TGF-β (**B**), ROR-γ (**C**), STAT3 (**D**), IL-17A (**E**), and IL-23 (**F**) in zones Z3, Z2, and Z1 of acini and PT in the liver parenchyma of 21 patients with fatal YF compared to the 05 normal controls. Z3: Pericentral zone; Z2: Midzonal zone; Z1: Periportal zone; PT: Portal tract. Tukey test *** *p* < 0.0001; ANOVA *** *p* < 0.0001.

Characteristic brown areas around or inside the cytoplasm, particularly in cells that comprise the inflammatory infiltrate, were observed by immunohistochemical staining. Immunolabeling revealed the presence of the cytokines IL-6, IL-17A, IL-23, and TGF-β, as well as the transcription factors ROR-γ and STAT3 (Table 3; Figure 2 and Figure 3) in the hepatic acinus and the PT. The expression of the Th17 markers were compared in Z3, Z2, Z1, and PT between the YF-infected samples and the control samples. The expression of these markers was significantly more intense in Z2 among the YF samples than among the control samples (*p* < 0.0001; Figure 4). The mean and standard deviation for each of the markers in both the YF-infected and control samples were determined (Figure 4 and Table 4).

## 4. Discussion

Historically, YF has been considered to be an infectious haemorrhagic disease with a major impact on public health. In this study, we attempted to describe the histopathological changes and the role of cellular and cytokine immune markers in the liver tissue of patients with YF who died of hepatic-renal failure [13,23,24,25].

The results obtained in this study are consistent with previous research, wherein mild or moderate inflammatory infiltrates were associated with injury to the midzone and the presence of Councilman bodies in hepatocytes (indicating apoptosis), followed by macro- or microvesicular steatosis and lytic or coagulative necrosis [5,11,12,13,24,25,26]. Frequently accompanying these changes are swelling, regeneration of hepatocytes, hyperplasia and hypertrophy of Kupffer cells, sinusoidal congestion, and haemorrhage of the parenchyma. Several human studies have revealed acute fulminant hepatitis with extensive acinar involvement, usually accompanied by a mild or moderate inflammatory infiltrate, in cases of severe YF infection.

Our findings demonstrated an increase in the expression of both Th17 cytokines and transcription factors in the liver samples of patients with fatal YF compared to controls. This increased expression was observed mainly in Z2, followed by Z3, Z1, and PT (*p* > 0.0001).

TGF-β is a cytokine that has been investigated in the immunopathogenesis of YF. It is a dichotomous and pleiotropic cytokine that exerts multiple immune functions [5,12,14,26]. Thus, in addition to being considered a potent inducer of apoptosis, it is also involved in the regulation of tissue repair [26,27,28]. TGF-β streamlines the tissue repair process by the activation of signalling pathways in response to being triggered by the SMADs. In the studied fulminant cases, cell damage occurred due to inflammation and tissue hypoxia. Kupffer cells attempt to reverse the tissue damage via transition to the M2 phenotype and by the activation of an alternate pathway [29,30,31,32]. Therefore, it is worth noting that, in this scenario, TGF-β can induce the production of several components, including collagen I, II, and III, undulin, fibronectin, laminin, elastin, proteoglycans, and hyaluronan, to regulate the integrity of the extracellular matrix; thus, it plays an important role in tissue remodelling [33,34].

IL-6 is a pleiotropic cytokine that acts together with TGF-β in the differentiation of naïve T cells into Th17 cells. IL-6 can also stimulate myofibroblasts and hepatocytes to synthesise collagen, which is important for the re-formation of the reticular fibres that make up the sinusoid cords [17,35,36]. One of the central properties of IL-6 against flaviviruses is the potentiation of the acute inflammatory response with an effect on the endothelium. In cases suggestive of fulminant hepatitis caused by dengue virus, it has been shown that cytokines can orchestrate an increased expression of IL-8, which serves as a chemotactic factor attracting leukocytes to the infection foci. In addition, in cases of haemorrhage, vasculopathy, and thrombocytopenia, cytokine expression is positively correlated with endothelial injury. This is primarily triggered by the action of IL-6 as well as other adhesion molecules and pro- and anti-inflammatory cytokines [37,38,39]. Haemorrhagic necrosis is a key phenomenon found in the hepatic parenchyma of the studied cases; it indicates the contribution of IL-6 and other immune factors, in situ, in generating a harmful environment in the hepatic parenchyma of patients with fatal YF.

Our study demonstrated the presence of transcription factors, such as STAT3, in the liver tissue of cases with fatal YF. STAT3 has been shown to play a role in pro- or anti-inflammatory responses in viral liver diseases, such as hepatitis B, which can lead to fibrosis [40]. In previous studies regarding flaviviruses, it was observed that non-structural proteins, such as NS1 of DENV-2, interact with transcription factors, such as STAT3 and ROR-γ, to increase the expression of IL-6 and TNF-α, respectively, thereby aggravating the inflammatory process in haemorrhagic conditions. These findings highlight the role of STAT3 and ROR-γ in the induction of the Th17 response [41,42].

IL-17A is highly expressed in cases with fatal YF. Our data indicate that IL-17A is involved in the secretion of cytokines and chemokines with an effect on the recruitment and activation of neutrophils and their migration to the site of infection. This explains the presence of neutrophilic infiltration, especially close to the foci of lytic necrosis, thus reinforcing the role of Th17 responses in the evolution of YFV infection [22,43]. Other studies involving DENV reported that, in the absence of IL-22, IL-17A could be instrumental in increasing the expression of CXCL1/KC, CCL5, IFN-γ, IL-6, and caspase 3; NK cell activity; and the recruitment of CD8-T cells, indicating the potential role of IL-17A as a key cytokine in the cascade of inflammatory responses and cell death in the liver, and thus, in the pathogenesis of YF [44].

IL-23 is considered to stabilise the differentiation and maturation of Th17 cells [44]. In a study investigating the mechanisms of cell damage in the central nervous system of fatal cases with microcephaly caused by Zika virus, increased expression levels of both IL-17A and IL-23 were observed, as well as enhanced Th1–mediated inflammation [20].

In a study on the hepatitis B virus, IL-23 modulated the M1 phenotype and induced the production of IL-1β, IL-6, TNF-α, and IFN-γ, and consequently, ROS [45,46]. In such instances, the presence of IL-23 would be necessary to induce the production of VEGF by activating the JAK/STAT3 pathway.

## 5. Conclusions

The results indicate that the in situ Th17 profile contributes markedly to the exacerbation of the inflammatory response observed in the livers of patients with fatal YF. The inflammatory events in the hepatic parenchyma results in cell damage and cell death and leads to endothelial injury, which favours the occurrence of the typical haemorrhagic diathesis phenomena observed in patients with YF.

## Figures and Tables

**Table 1:** Characteristics demographic of 21 Brazilian yellow fever patients.

Case	Sex	Age (Years)	Geographic Location
1	F	03	GO
2	F	36	GO
3	M	56	DF
4	M	48	MT
5	F	50	MG
6	M	38	MG
7	-	-	-
8	M	39	MG
9	M	28	MG
10	F	-	MG
11	M	33	MG
12	-	-	-
13	M	23	GO
14	M	54	GO
15	M	32	DF
16	M	24	GO
17	F	63	GO
18	M	55	DF
19	M	46	DF
20	M	35	DF
21	M	-	-

Legends: (-): not available; M: male; F: female; GO: Goias state; DF: Federal district; MT: Mato Grosso state; MG: Minas Gerais state.

**Table 2:** Semi-quantitative analysis of morphological changes to the hepatic acinus (Z3, Z2, Z1) and the portal tract (PT) in the liver of 21 patients with fatal YF and 05 normal controls. (+) Present, light intensity; (++) Present, moderate intensity; (+++) Present, high intensity; (-) absent.

Morphological Changes	Z3	Z2	Z1	PT
Cell swelling	2++	+++	+++	-
Macrovesicular steatosis	+	++	+	-
Microvesicular steatosis	++	+++	++	-
Lytic necrosis	+	++	++	-
Coagulative necrosis	+	+	+	-
Councilman bodies	+++	+++	+++	-
Kupffer cell hyperplasia	+	++	++	-
Kupffer cell hypertrophy	+	++	++	-
Oedema	-	-	-	++
Congestion	-	-	-	++
**Inflammatory Infiltrate**				
Lymphocytes	+	++	+	++
Neutrophils	+	+	+	+
Plasma cells	+	++	+	++
Eosinophils	+	+	+	+
Macrophages	-	-	-	++
Sinusoidal endothelial alteration	++	++	++	-
Alteration centrilobular vein	+++	+	+	-
Sinusoidal congestion	+	++	+	-
Cholestasis	+	+	+	+
Sinusoidal dilatation	+	+	+	-
Portal vein alteration	-	-	-	++
Portal artery alteration	-	-	-	++
Bile canaliculus alteration	-	-	-	+
Limiting plaque injury	-	-	-	+

Z3: Pericentral zone; Z2: Midzonal zone; Z1: Periportal zone; PT: Portal tract.

**Table 3:** Quantitative analysis (mean ± standard deviation) of Th17 markers in the liver parenchyma (Z3, Z2, Z1, and PT) in 21 patients with fatal YF and 05 normal controls.

Markers	Z3 (Cells/mm^2^)	Z2 (Cells/mm^2^)	Z1 (Cells/mm^2^)	PT (Cells/mm^2^)	ANOVA (*p* ≤ 0.05)
IL-17A Control	162.20 ± 18.81 42.56 ± 7.72	230.90 ± 22.63 55.36 ± 4.32	145.80 ± 13.36 36.80 ± 8.23	128.10 ± 7.20 37.76 ± 3.85	***
Tukey (*p* ≤ 0.05)	***	***	***	***	
IL-23	140.80 ± 12.77	213.80 ± 17.54	124.60 ± 10.05	109.80 ± 7.46	***
Control	66.24 ± 39.15	71.36 ± 52.59	62.72 ± 38.77	44.16 ± 9.37	
Tukey (*p* ≤ 0.05)	***	***	***	***	
IL-6	121.10 ± 15.78	182.60 ± 17.91	104.80 ± 12.64	94.32 ± 14.69	***
Control	45.44 ± 23.61	56.96 ± 25.21	36.80 ± 14.49	33.92 ± 4.58	
Tukey (*p* ≤ 0.05)	***	***	***	***	
STAT3	133.20 ± 11.15	176.90 ± 15.22	115.70 ± 8.95	101.40 ± 8.82	***
Control	28.16 ± 3.50	29.76 ± 2.42	25.92 ± 2.62	24.96 ± 2.90	
Tukey (*p* ≤ 0.05)	***	***	***	***	
RORγ	124.30 ± 11.20	185.10 ± 15.42	113.40 ± 7.67	96.15 ± 10.67	***
Control	29.44 ± 2.67	32.32 ± 3.46	25.28 ± 3.46	26.88 ± 4.71	
Tukey (*p* ≤ 0.05)	***	***	***	***	
TGF-β	135.90 ± 9.64	207.40 ± 17.86	119.20 ± 10.03	115.90 ± 12.10	***
Control	63.68 ± 42.85	72.32 ± 48.31	49.60 ± 29.18	35.52 ± 9.28	
Tukey (*p* ≤ 0.05)	***	***	***	***	

Z3: Pericentral zone; Z2: Midzonal zone; Z1: Periportal zone; PT: Portal tract. ANOVA one-way *** *p* < 0.0001; Tukey *** *p* < 0.0001.

**Table 4:** Comparative analysis (mean ± standard deviation) of the expression of Th17 markers in 21 YF patients between the different compartments studied.

Markers	Z2 vs. Z1 (Cells/mm^2^)	Tukey *p*	Z2 vs. Z3 (Cells/mm^2^)	Tukey *p*	Z2 vs. PT (Cells/mm^2^)	*p* Tukey
IL-17A	230.90 ± 22.63 vs.145.80 ± 13.36	***	230.90 ± 22.63 vs.162.20 ± 18.81	***	230.90 ± 22.63 vs.128.10 ± 7.200	***
IL-23	213.80 ± 17.54 vs.124.60 ± 10.05	***	213.80 ± 17.54 vs.140.80 ± 12.77	***	213.80 ± 17.54 vs.109.80 ± 7.461	***
IL-6	182.60 ± 17.91 vs.104.80 ± 12.64	***	182.60 ± 17.91 vs.121.10 ± 15.78	***	182.60 ± 17.91 vs.94.32 ± 14.69	***
STAT3	176.90 ± 15.22 vs.115.70 ± 8.956	***	176.90 ± 15.22 vs.133.20 ± 11.15	***	176.90 ± 15.22 vs.101.40 ± 8.82	***
RORγ	185.10 ± 15.42 vs.113.40 ± 7.67	***	185.10 ± 15.42 vs.124.30 ± 11.20	***	185.10 ± 15.42 vs.96.15 ± 10.67	***
TGF-β	207.40 ± 17.86 vs.119.20 ± 10.03	***	207.40 ± 17.86 vs.135.90 ± 9.646	***	207.40 ± 17.86 vs.115.90± 12.10	***

Z3: Pericentral zone; Z2: Midzonal zone; Z1: Periportal zone; PT: Portal tract. Tukey test *** *p* < 0.0001.

## Data Availability

The database used and/or analysed during the current study is not publicly accessible but can be available, upon reasonable request, from the corresponding authors.
